# Functions of exosome-derived noncoding RNA-encoded polypeptides/proteins in human diseases

**DOI:** 10.1016/j.gendis.2026.102128

**Published:** 2026-03-05

**Authors:** Xinyuan Feng, Meihuan Chen, Dahang Ye, Ying Zhu, Liangpu Xu, Hailong Huang

**Affiliations:** a Medical Genetic Diagnosis and Therapy Center of Fujian Maternity and Child Health Hospital College of Clinical Medicine for Obstetrics & Gynecology and Pediatrics, Fujian Medical University, Fujian Provincial Key Laboratory of Prenatal Diagnosis and Birth Defect, Fuzhou, Fujian 350001, China; b Key Laboratory of Clinical Laboratory Technology for Precision Medicine (Fujian Medical University), Fujian Province University, Fuzhou, Fujian 350122, China

**Keywords:** Biomarkers, EDNEPs, Exosomes, ncRNAs, Therapeutic target

## Abstract

Exosomes are nanoscale vesicles that are ubiquitously present in biological fluids and exhibit remarkable stability, carrying a diverse array of biologically active molecules, including various RNAs, DNAs, and proteins. Exosomes are crucial mediators of intercellular signaling and exert extensive regulatory effects on nearly all physiological and pathological processes in the body. Traditionally, noncoding RNAs (ncRNAs) were deemed incapable of encoding proteins, but recent studies have demonstrated that noncoding RNAs harbor a substantial number of open reading frames, which possess the potential to encode peptides/proteins that are implicated in the pathogenesis and progression of various diseases. This review aims to comprehensively summarize the roles and mechanisms of exosome-derived ncRNA-encoded polypeptides/proteins (EDNEPs) in common diseases across nine major physiological systems, including the hematological, motor, digestive, respiratory, urinary, endocrine and reproductive, circulatory, immune, and nervous systems. Additionally, this review seeks to provide references for the clinical application of EDNEPs as diagnostic and prognostic biomarkers, promising therapeutic agents, and potential therapeutic targets.

## Introduction

Exosomes are extracellular vesicles with a diameter ranging from 30 to 150 nm. These vesicles are present in virtually all body fluids, where they carry a rich array of bioactive molecules. They play crucial roles in cell-to-cell communication, thereby regulating a wide spectrum of physiological and pathological processes within the organism. Under normal physiological conditions, the vast majority of cells can continuously secrete exosomes that are loaded with key signaling molecules and metabolites. These exosomes regulate cell growth, differentiation, apoptosis and metabolism and are involved in cellular adaptation to environmental changes and stress responses. Additionally, exosomes facilitate intercellular exchange of substances and removal of metabolic wastes, thereby maintaining intracellular homeostasis. In addition, certain specialized cell types, such as immune cells, can secrete exosomes that carry immunoregulatory molecules, including cytokines and antigens, to modulate the activity and function of immune cells.

Noncoding RNAs (ncRNAs) mainly include microRNAs (miRNAs), long noncoding RNAs (lncRNAs), and circular RNAs (circRNAs). MiRNAs can regulate the expression of target genes by binding to target messenger RNA (mRNA) and inducing mRNA silencing. CircRNAs and lncRNAs have similar regulatory functions. Both can act as miRNA sponges to modulate gene expression and can also regulate gene expression through interactions with RNA-binding proteins. Historically, ncRNAs were considered to lack coding potential. However, recent studies have identified open reading frames (ORFs) within their sequences, which enable them to encode polypeptides or proteins. Current research into the coding capabilities of ncRNAs is opening up new avenues for disease diagnosis and treatment. Moreover, polypeptides/proteins encoded by exosomal ncRNAs can influence cell growth, proliferation, and metabolism via a variety of mechanisms, thereby impacting disease progression.

This review systematically summarizes the biogenesis and functions of exosomes, the potential of ncRNA-encoded polypeptides/proteins, and the roles and mechanisms of exosome-derived ncRNA-encoded polypeptides/proteins (EDNEPs) in various common diseases across different systems. Our study aims to provide references for the development of EDNEPs as novel biomarkers, therapeutic agents, and therapeutic targets in human diseases.

## Exosomes

### Production of exosomes

Exosomes are extracellular vesicles (EVs) with a diameter ranging from 30 to 150 nm and a density of 1.1–1.2 g/mL, which are released into the microenvironment by cells under normal conditions or pressure. Exosomes are present in virtually all body fluids, including blood, urine, breast milk, and saliva.^1^^,^^2^

**Figure 1:**
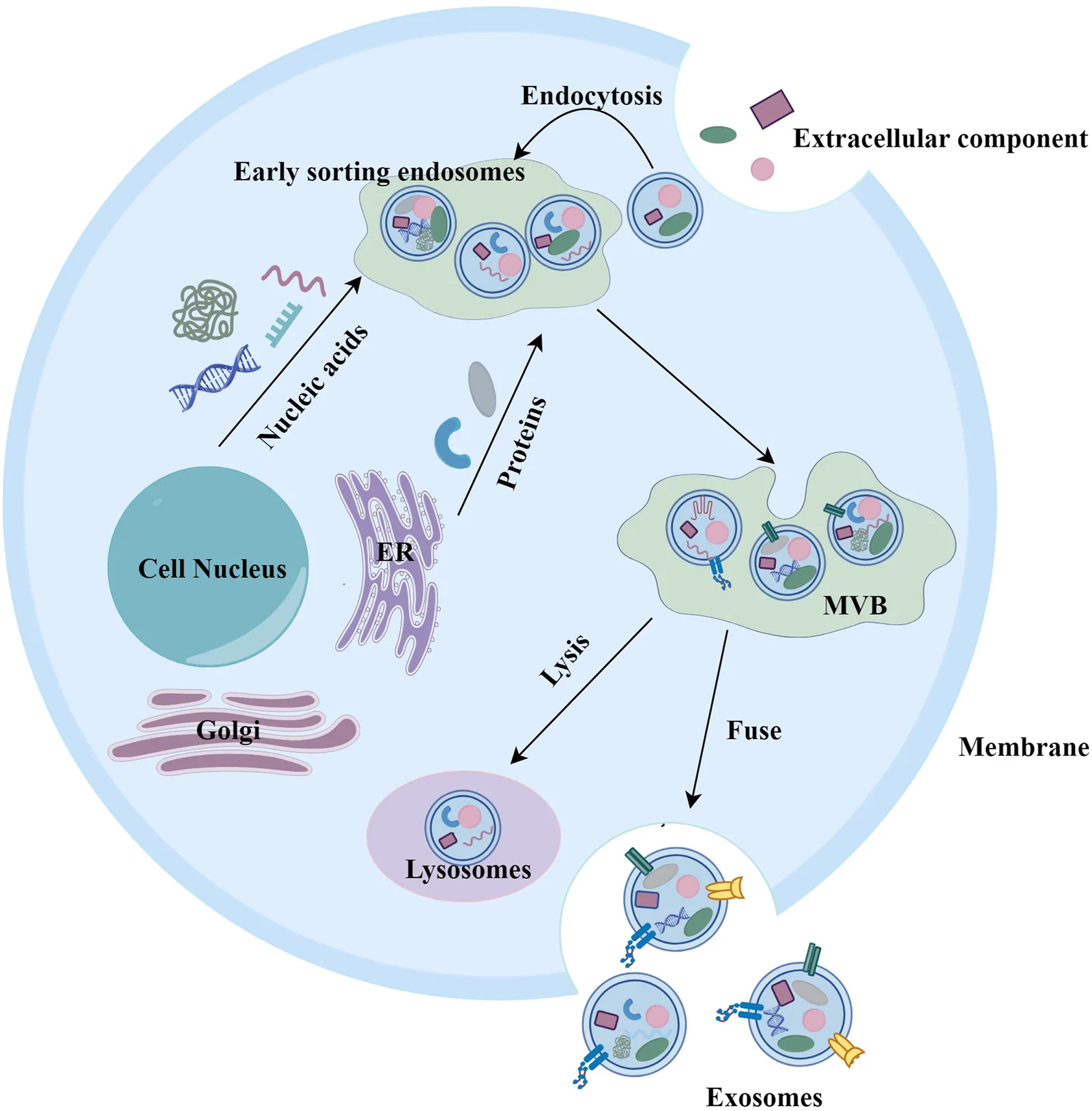
The process of exosome formation. First, the cell membrane invaginates to form early sorting endosomes, and nucleic acids and proteins are released into the early sorting endosomes. Subsequently, some membrane vesicles on the endosomal membrane sprout inward to form multivesicular bodies (MVBs). Finally, the vesicles in the MVB are released into the extracellular environment to form exosomes.

Exosomes carry a diverse array of biologically active molecules, including ncRNAs, DNAs, lipids, metabolites, and both structural and secretory proteins. They play a crucial role in cell–cell communication by transferring these molecules from donor cells to recipient cells, thereby modulating the regulation of a wide range of physiological and pathological processes within the organism. Exosome formation is divided into three processes (Fig. 1): initially, cells take up extracellular substances through endocytosis to form early sorting endosomes; subsequently, vesicles on the endosomal membrane bud inward and encapsulate the endosomal material to form multiple vesicles, and the early endosomes transform into multivesicular bodies (MVBs); finally, the MVBs fuse with the cellular membrane and release the intracellular vesicles into the extracellular environment to form an exosome.^3^ Exosome formation is precisely regulated by both endosomal sorting complexes required for transport (ESCRT)-dependent and nondependent pathways.

### Communication of exosomes

**Figure 2:**
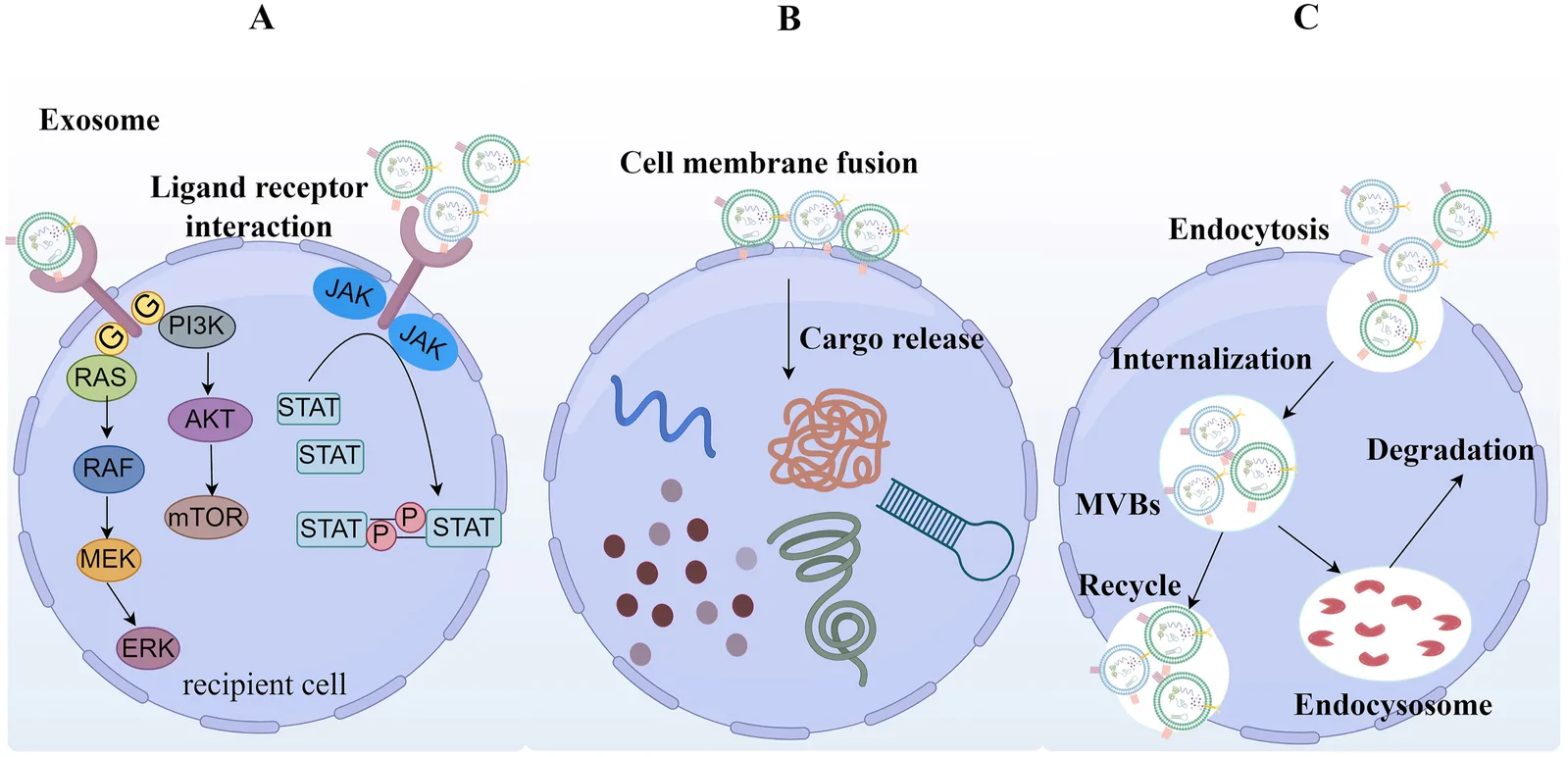
The intercommunication between exosomes and receptor cells. **(A)** Transmembrane proteins in exosomes bind directly to signaling receptors in recipient cells and activate intracellular signaling cascade signaling pathways, such as JAK/STAT, MAPK, and PI3K/AKT/mTOR. **(B)** Exosomes fuse with the plasma membrane of the recipient cells and release contents such as proteins, lipids, and nucleic acids into the cells. **(C)** Exosomes are internalized in recipient cells, and some of them fuse to undergo endocytosis, transcytosis and release into neighboring cells. Others fuse to form endocytosis and mature into lysosomes and are finally degraded.

The intercommunication between exosomes and receptor cells occurs through three primary mechanisms. First, transmembrane proteins present in exosomes directly bind to signaling receptors on recipient cells, thereby activating intracellular signaling cascade pathways^4^ (Fig. 2A). Second, exosomes can fuse with the plasma membrane of recipient cells, releasing their contents into the cells^5^ (Fig. 2B). Third, upon internalization into recipient cells, some exosomes may merge with endosomes and undergo transcytosis for release into neighboring cells, while others may fuse to form endosomes that mature into lysosomes and are subsequently degraded^6^ (Fig. 2C). Exosomes have emerged as ideal therapeutic carriers due to their low toxicity, high permeability, enhanced biocompatibility, nonimmunogenicity, and specific targeting capabilities for various cells.

### Functions of exosomes

#### Mediating intercellular signal transduction and cell–cell communication

Exosomes, as carriers of various cellular components, including proteins, lipids, and nucleic acids, play a pivotal role in intercellular communication by interacting with target cells via endocytosis.^7^ The nucleic acid substances transported by exosomes, particularly mRNA and miRNA, are central to their mediated signal transduction pathways. Upon incorporation into recipient cells, the mRNAs carried by exosomes are translated into functional proteins. This process directly modulates cellular functional properties, inducing specific metabolic changes or alterations in cellular morphology. On the other hand, the miRNAs carried by exosomes can inhibit mRNA translation through complementary pairing with their target mRNAs, thereby regulating gene expression in recipient cells. Additionally, proteins transported by exosomes play a crucial role in cellular communication by interacting with the receptors of target cells, thereby activating or inhibiting intracellular signaling pathways. More importantly, exosomes are involved in the amplification and propagation of signals during intercellular communication. Exosomes secreted by a donor cell can influence multiple target cells, and the target cells can secrete additional exosomes upon receiving the signals, thereby further propagating the signals. This process establishes a complex and coordinated signaling network within local tissues or even throughout the entire body, facilitating the synergistic response of cells to physiological demands and pathological alteration.^8^^,^^9^

### Modulating immune response

Exosomes play pivotal roles in immune regulation, encompassing antigen presentation, immune activation, suppression and tolerance. They contain major histocompatibility complex class II (MHC II) antigenic peptides, which can be directly presented to specific T cells, thereby inducing their activation.^10^ Exosome-derived immunogenic peptides can activate immature mouse dendritic cells and indirectly activate antigen-presenting cells (APCs), thereby inducing the proliferation of specific CD4+ T cells.^11^ The miRNAs in exosomes can modulate gene expression and signaling pathways within recipient cells, thereby controlling both innate and adaptive immune responses and influencing the maturation of dendritic cells.^12^ In mice, exosomes stimulate adaptive immune responses, which include the activation of dendritic cells, the uptake of exosomal genomic DNA derived from breast cancer cells, and the activation of the cGAS-STING signaling pathway, leading to antitumor responses.^13^

### Maintaining cell homeostasis

Exosomes mitigate intracellular stress and preserve cellular homeostasis by eliminating damaged or toxic substances, including proteins, lipids, and nucleic acids. When the secretion of exosomes is impeded, the accumulation of nuclear DNA in the cytoplasm triggers the activation of the cytoplasmic DNA sensing pathway. This exacerbates the innate immune response and leads to reactive oxygen species (ROS)-dependent DNA damage response, thereby inducing senescence-like cell cycle arrest or apoptosis in human cells.^14^ Furthermore, exosomes modulate cellular homeostasis by facilitating the elimination of misfolded or degraded RNA products as well as toxic RNA molecules. Studies have demonstrated that cells maintain intracellular RNA homeostasis by expelling various RNAs via exosomes. Additionally, proteins involved in RNA processing are prevalent in exosomes, and the half-life of secreted RNAs is nearly twice as long as that of intracellular mRNAs.^15^, ^16^, ^17^

### Potential of ncRNA-encoded polypeptides/proteins

Traditionally, ncRNAs were deemed nontranslatable and were referred to as the “dark matter” of genomics. However, recent research has revealed that ncRNAs contain ORFs capable of encoding polypeptides/proteins.^18^

**Figure 3:**
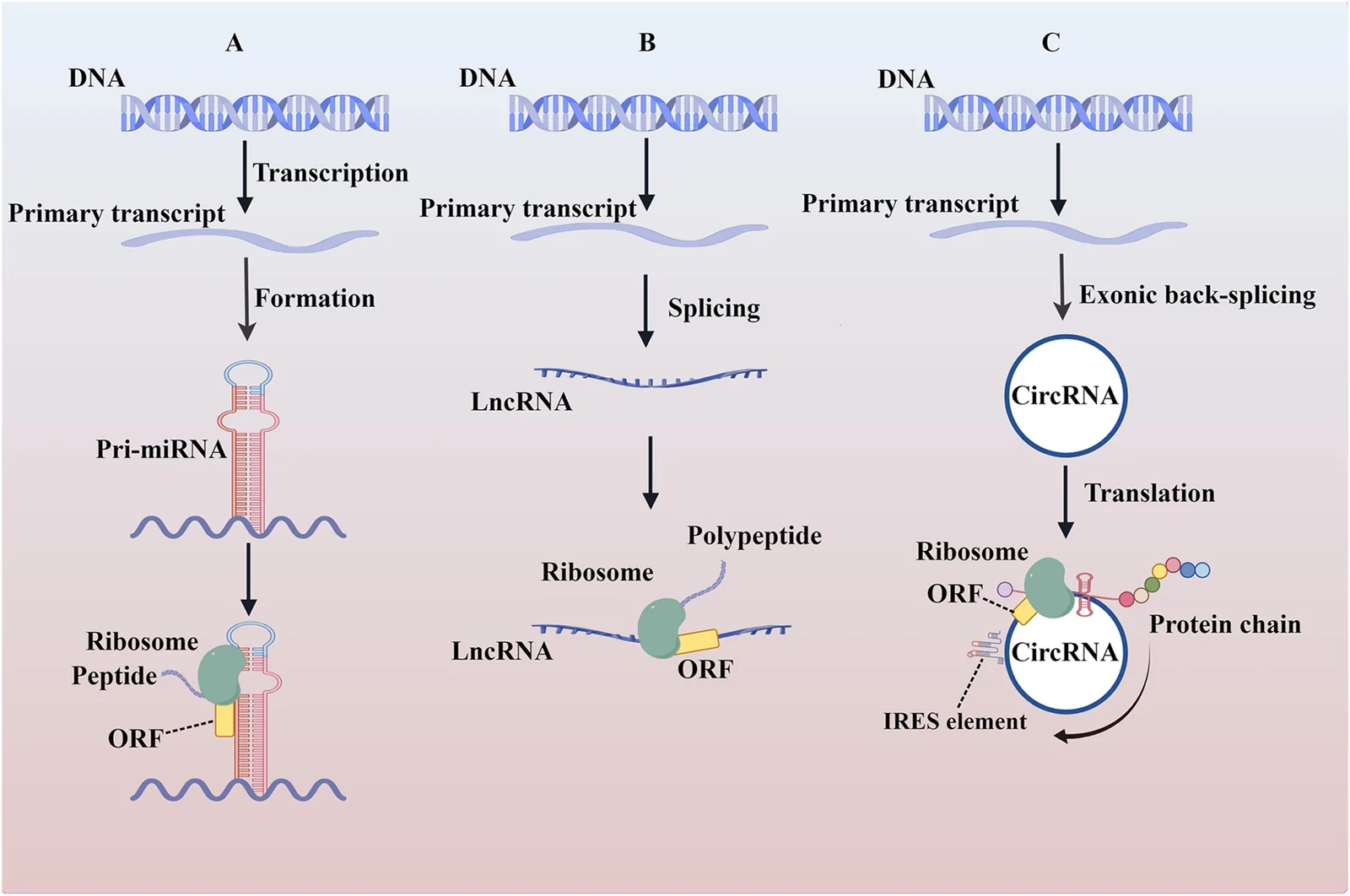
Formation of polypeptides/proteins encoded by ncRNAs. The DNA sequences are transcribed into the primary transcripts, which are then formed into pri-miRNA (A), spliced into lncRNA (B) and back-spliced into circRNA (C). Sequences of pri-miRNAs and lncRNAs contain unique open reading frames (ORFs) that are recognized by ribosomes and translated into polypeptides. The ORFs in circRNA sequences are recognized by ribosomes through internal ribosomal entry sites (IRESs) and translated into polypeptides.

Mature miRNAs are derived from primary transcripts (pri-miRNAs) through a series of shearing and processing steps mediated by nucleases. Some pri-miRNA sequences encompass short ORFs that can be translated into polypeptides encoded by miRNA primary transcripts (miPEPs)^19^^,^^20^ (Fig. 3A). LncRNAs are produced through DNA transcription, and certain lncRNA sequences contain ORFs, which can be recognized and translated by ribosomes to generate peptides under specific conditions^21^ (Fig. 3B). CircRNAs are a class of circular ncRNAs formed through reverse splicing, and some circRNA sequences possess internal ribosome entry sites (IRES) and ORFs that can be recognized by ribosomes, leading to the translation and production of polypeptides^22^ (Fig. 3C).

### miRNAs

A growing body of research indicates that ribosomes can identify ORFs on pri-miRNAs and associated translation initiation signals, thereby initiating the translation process. Transfer RNA (tRNA) transports the corresponding amino acids in accordance with the codon sequence on the ORFs to synthesize miPEPs. Typically, miPEPs are short in length and exhibit tissue- and cell-specific expression.^23^, ^24^, ^25^ The primary function of miPEPs is to enhance the accumulation of mature miRNAs by positively regulating the transcription of their corresponding pri-miRNAs, thereby augmenting the regulatory impact of miRNAs on target genes. For instance, miPEP171b enhances the accumulation of endogenous miR171b in alfalfa plants, while miPEP172c increases the accumulation of miR172c by promoting the expression of pri-miR172c in soybeans.^23^

Moreover, miPEPs are involved in intracellular signal transduction pathways and are capable of interacting with other proteins to facilitate the transmission of both intracellular and extracellular signals, thereby regulating cellular physiological functions.^25^^,^^26^ Furthermore, miPEPs may exert influence on cellular processes such as proliferation, differentiation, and apoptosis and play a role in determining cell fate by modulating the expression of critical genes.^20^^,^^27^ For instance, miPEP133, a novel microprotein encoded by pri-miR-34a, interacts with mitochondrial heat shock protein 70 (HSP70). This interaction regulates mitochondrial morphology and function, slows migration and invasion, and induces cell cycle arrest, growth inhibition, and apoptosis in cancer cells.^20^

### LncRNAs

Recent studies have demonstrated that functional micropeptides encoded by lncRNAs are primarily associated with several key factors.^28^, ^29^, ^30^ Initially, molecular techniques such as ribosome-imprinted sequencing (Ribo-seq) and mass spectrometry analysis have revealed that certain lncRNAs contain ORFs, which confer the capacity to encode small peptides or micropeptides upon association with ribosomes.^31^ Second, lncRNAs acquire coding potential through alternative splicing, which generates numerous transcripts harboring intact ORFs. Third, epigenetic modifications, including DNA methylation, histone modification, and chromatin remodeling, influence the transcription, expression, structure and function of lncRNAs, thereby enabling them to gain encoding capabilities. LncRNAs typically encode short peptides consisting of fewer than 100 amino acids, which can be expressed in specific tissues and cells.^32^^,^^33^

On the one hand, polypeptides encoded by lncRNAs can bind to mRNAs, thereby affecting mRNA stability through altering mRNA structure or interacting with proteins involved in mRNA degradation. On the other hand, lncRNA-encoded polypeptides can also participate in processes such as alternative splicing, gene transcriptional regulation, and protein phosphorylation or degradation.^34^ Furthermore, lncRNA-encoded peptides are involved in the modulation of cellular physiology. Myoregulin, a small peptide encoded by lncRNA in mice, regulates the transport of calcium ions within the sarcoplasmic reticulum, which plays a crucial role in muscle growth, development, and metabolism processes.^35^ LncRNA-encoded peptides contribute to the development and progression of cancers and may serve as valuable targets for therapeutic intervention.^34^ For instance, the micropeptide SMIM30, encoded by LINC00998, facilitates the G1/S transition of the cell cycle by reducing cytoplasmic calcium concentrations. The peptide CASIMO1, encoded by lncRNA, interacts with squalene epoxidase to modulate lipid droplet aggregation and the proliferation of breast cancer cells.^36^

### CircRNAs

Since circRNAs lack a 5′ terminal 7-methylguanosine cap structure and a 3′ terminal poly(A) tail, they are capable of translating peptides solely through cap-independent mechanisms. CircRNAs encode polypeptides through the following mechanisms. First, the presence of highly conserved ORFs in circRNAs facilitates polypeptide translation. This process is mediated by IRES elements that recruit ribosomes and thereby initiate translation. Second, circRNAs initiate polypeptide translation by interacting with N6-methyladenosine (m6A)-binding proteins and translation initiation factors via their m6A modification sites. Third, circRNAs contain only an initiation codon (AUG) without a termination codon, allowing ribosomes to undergo continuous rolling translation within the ORFs, resulting in the production of polypeptides with numerous repeated sequences.^34^

The polypeptides encoded by circRNAs are relatively short, exhibiting high specificity and stability, as well as low toxicity.^37^ They can be found in various cellular compartments, including the nucleus, cytoplasm, and cell membrane, and as secreted proteins. CircRNA-encoded peptides exert diverse functions in both physiological and pathological cellular processes, particularly within tumors, where they can function as either oncogenes or tumor suppressors.^22^ For instance, functional peptides derived from circ-FBXW7, circ-SHPRH, and circPINTexon2 could inhibit the proliferation and tumorigenicity of glioma cells.^38^, ^39^, ^40^

### Regulatory roles of EDNEPs in disease progression

**Figure 4:**
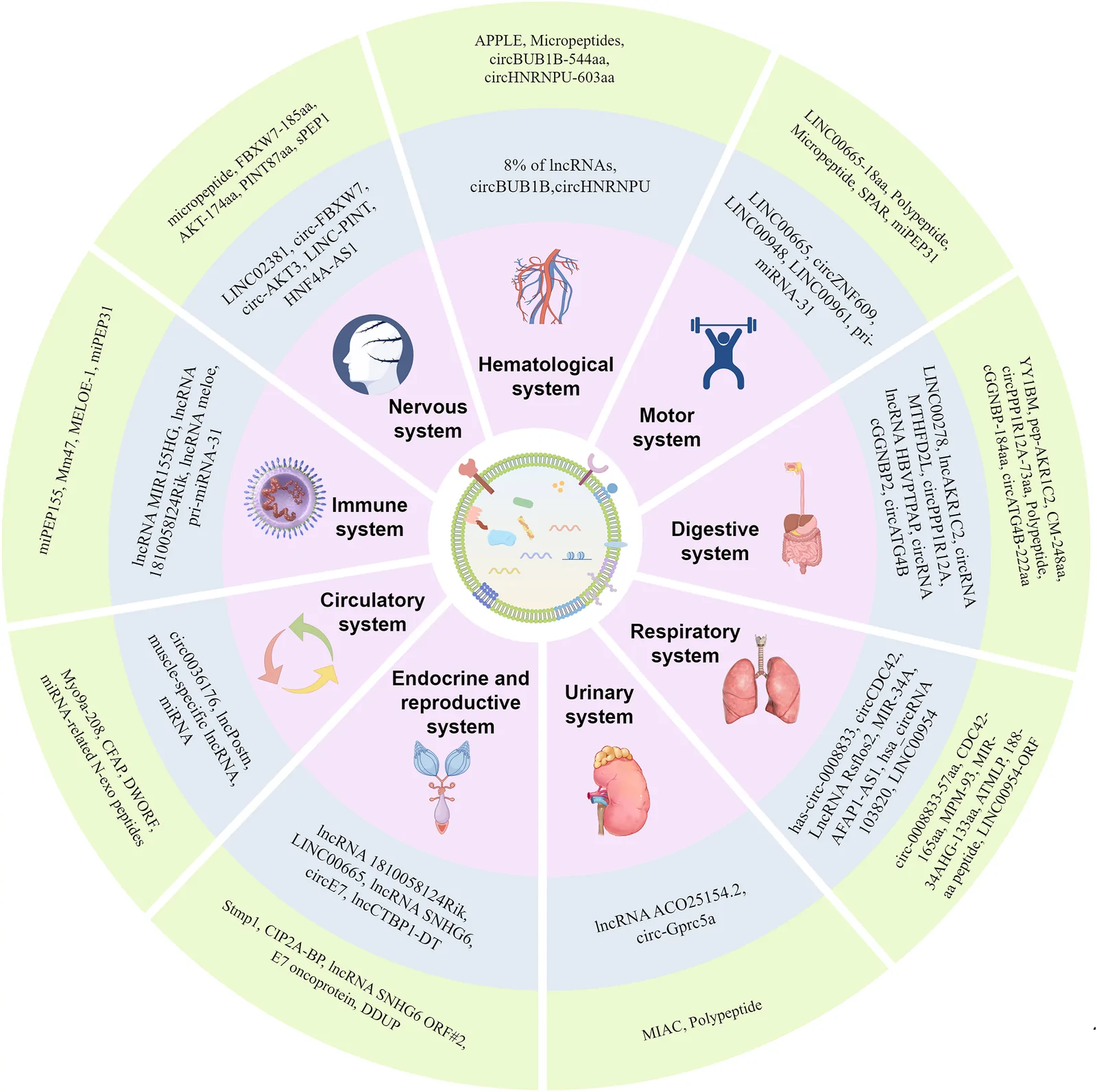
A schematic representation of exosome-derived ncRNAs and their encoded polypeptides in nine human systems. The diagram is organized into three concentric layers: the innermost ring (systems), the middle ring (ncRNAs), and the outermost ring (encoded peptides).

An increasing body of research indicates that EDNEPs play a role in disease development, encompassing the extensive regulation of cellular processes such as proliferation, invasion, and metastasis in cancers, as well as significant effects on inflammatory responses, tissue repair, and immune regulation in nonneoplastic diseases. The regulatory roles of EDNEPs in human diseases of nine systems (hematological, motor, digestive, respiratory, urinary, endocrine and reproductive, circulatory, immune, and nervous) afford a novel avenue for a better understanding of disease pathogenesis. Additionally, they offer innovative perspectives for the diagnosis and treatment of these conditions. Figure 4 provides a summary of exosome-derived ncRNAs and their encoded polypeptides in various systems.

### Hematologic system

EDNEPs in the hematologic system have been predominantly studied in leukemia and multiple myeloma (MM) (Table 1). In acute myeloid leukemia (AML), Sun^41^ et al discovered that lncRNAs are highly enriched in exosomes by leveraging ribosomal sequencing, mass spectrometry analysis, and RNA sequencing technologies. A peptide named APPLE, encoded by lncRNAs, is highly expressed in ribosomes and interacts with eIF4G to facilitate mRNA cyclization and the assembly of eIF4G initiation complexes, thereby promoting specific oncogenic translational processes that sustain the malignant phenotype of AML. In chronic myeloid leukemia (CML), Bánfai^42^ et al conducted RNA sequencing and tandem mass spectrometry analysis on K562 and Gm12878 (lymphoblasts) cell lines. Their analysis revealed that approximately 8% of lncRNAs are translatable into micropeptides. However, this study only confirmed the existence of micropeptides and lacked in-depth research on the specific mechanisms of these micropeptides in the drug resistance of CML cells. In MM, Tang^43^ et al demonstrated that BUB1 mitotic checkpoint serine/threonine kinase B (BUB1B) is selectively incorporated into exosomes within a specific intracellular molecular context. The circBUB1B-encoded peptide circBUB1B-544aa is secreted into the bone marrow microenvironment, where it induces chromosomal instability by phosphorylating centrosomal protein 170 (CEP170). This process promotes proliferation and enhances the drug resistance of MM cells. Furthermore, utilizing a heterogeneous nuclear ribonucleoprotein U (HNRNPU) antibody and mass spectrometry, researchers identified a peptide encoded by circHNRNPU, specifically circHNRNPU-603aa, that was found to contain an RNA-binding arginine-glycine-glycine (RGG) motif, which is capable of modulating exon skipping in S-phase kinase-related protein 2 (SKP2), thereby promoting the onset and progression of MM^44^. Therefore, by regulating translation, inducing chromosomal instability, and modulating gene splicing, EDNEPs have been shown to drive malignant progression and drug resistance in hematological malignancies.

**Table 1:** The roles and mechanisms of EDNEPs in human diseases of the hematological and motor systems.

Systems	Diseases	ncRNAs	Encoded polypeptides/proteins	Functions	Mechanisms	References
Hematological system	AML	Unclear	APPLE	Maintains the malignant phenotype of AML	Interacts with eIF4G to promote specific oncogenic translational processes	Sun et al^[^^41^^]^
	CML	8% of lncRNAs	Micropeptides	Unclear	Unclear	Bánfai et al[^42^]
	MM	circBUB1B	circBUB1B-544aa	Promotes proliferation and drug resistance of MM cells	Induction of chromosomal instability by phosphorylation of CEP170	Tang et al^[^^43^^]^
	MM	circHNRNPU	circHNRNPU-603aa	Promotes the occurrence and development of MM	Regulation of SKP2 exon skipping	Tang et al^[^^44^^]^
Motor system	OS	LINC00665	LINC00665-18aa	Inhibits OS cell viability, proliferation and migration *in vitro* and reduces tumor growth *in vivo*	Affects the transcriptional activity, nuclear localization, and phosphorylation of CREB1 and disrupts the interaction between CREB1 and RPS6KA3	Pan et al^[^^45^^]^
	DMD	circZNF609	Polypeptide	Regulates myogenic cell proliferation	Unclear	Legnini et al^[^^46^^]^
	Muscle	LINC00948	Micropeptide	Acts as a myoregulatory protein to regulate Ca^2+^ uptake and skeletal muscle contractility	Inhibits sarcoplasmic reticulum Ca^2+^-ATPase	Anderson et al^[^^35^^]^
	Muscle	LINC00961	SPAR	Inhibits muscle regeneration	Interacts with lysosomal v-ATPase to negatively regulate activation of mTORC1	Matsumoto et al^[^^47^^]^
	Muscle	pri-miRNA-31	miPEP31	Inhibits the vascular smooth muscle cell proliferation	Cooperation with transcription factor Trps1 to inhibit miR-31 expression	Jiang et al^[^^48^^]^

EDNEPs: exosome-derived ncRNA-encoded polypeptides/proteins, ncRNAs: noncoding RNAs; AML: acute myeloid leukemia; CML: chronic myeloid leukemia; MM: multiple myeloma; BUB1B: BUB1 mitotic checkpoint serine/threonine kinase B; CEP170: centrosomal protein 170; HNRNPU: heterogeneous nuclear ribonucleoprotein U; SKP2: S-phase kinase-related protein 2; OS: osteosarcoma; CREB1: cyclophosphoadenosine responsive progenitor protein 1; RPS6KA3: ribosomal protein S6 kinase A3; DMD: Duchenne muscular dystrophy; SPAR: small regulatory polypeptide of amino acid response; mTORC1: modulation of the activation of rapamycin complex 1; Trps1: transcriptional repressor GATA binding 1.

### Motor system

Current research on EDNEPs in the motor system has primarily concentrated on osteosarcoma (OS) and muscle diseases (Table 1). Pan^45^ et al discovered that the peptide LINC00665-18aa, encoded by LINC00665, attenuated the transcriptional activity, nuclear localization, and phosphorylation of cyclophosphoadenosine responsive element binding protein 1 (CREB1). Additionally, it disrupted the interaction between CREB1 and ribosomal protein S6 kinase A3 (RPS6KA3), which led to the inhibition of viability, proliferation and migration of OS cells *in vitro* and a reduction in tumor growth *in vivo*. In Duchenne muscular dystrophy (DMD), Legnini^46^ et al revealed that circ-ZNF609 associates with multiple ribosomes and undergoes translation in a splicing-dependent and cap-independent manner, thereby specifically regulating myogenic cell proliferation.

In addition, exosome-derived LINC00948 encodes a functional micropeptide that serves as a myoregulatory protein that inhibits sarcoplasmic reticulum Ca^2+^-ATPase, thereby modulating Ca^2+^ uptake and subsequently regulating skeletal muscle contractility.^35^ Exosome-derived LINC00961 encodes a functional micropeptide known as the “small regulatory polypeptide of amino acid response (SPAR)”, which is localized to late endosomes/lysosomes and interacts with lysosomal v-ATPase, thereby negatively modulating the activation of the mammalian target of rapamycin complex 1 (mTORC1). The downregulation of SPAR can effectively activate mTORC1 and facilitate muscle regeneration.^47^ Moreover, Jiang et al^48^ discovered that miPEP31, encoded by pri-miRNA-31, suppresses vascular smooth muscle cell proliferation through a cooperative mechanism with the transcription factor Trps1. These findings demonstrate that EDNEPs act by modulating key signaling pathways to control muscle cell proliferation, contraction, and regeneration in muscle diseases, revealing their crucial roles in the motor system.

### Digestive system

Recently, EDNEPs have been identified in several gastrointestinal malignancies (Table 2). In esophageal squamous cell carcinoma (ESCC), Wu^49^ et al reported that Y-linked LINC00278 in males with ESCC can translate the micropeptide YY1BM. Additionally, they found that cigarette smoking could diminish the m6A modification of LINC00278 and thus inhibit the translation of YY1BM. Functionally, YY1BM inhibits the interaction between the YY1 protein and the androgen receptor (AR), thereby reducing the expression of eEF2K. Conversely, the downregulation of YY1BM increases eEF2K expression and inhibits cell apoptosis, leading to enhanced adaptation to nutrient deprivation and promoting the progression of ESCC. In gastric cancer (GC), Zhu^50^ et al discovered that exosome-derived lncAKR1C2 secreted by GC cells encodes a microprotein named pep-AKR1C2 within lymphoendothelial cells. Further studies revealed that pep-AKR1C2 facilitates the expression of CPT1 by modulating YAP phosphorylation. This process enhances fatty acid oxidation and ATP production, thereby promoting the tube formation and migration of lymphatic endothelial cells. Consequently, it further advances lymph node metastasis in GC. Another study identified that the CM-248aa peptide, encoded by the circRNA MTHFD2L, competitively binds to the acidic structural domain of the nuclear gene SET and promotes the dephosphorylation of protein kinase B (AKT), extracellular signal-regulated kinase, and P65, thereby inhibiting the proliferation and metastasis of GC both *in vitro* and *in vivo*. Zheng^51^ et al discovered that circPPP1R12A is highly expressed in GC, and its encoded functional peptide, circPPP1R12A-73aa, drives the growth and metastasis of CC through the activation of the Hippo-YAP signaling pathway.

**Table 2:** The roles and mechanisms of EDNEPs in human diseases of the digestive and respiratory systems.

Systems	Diseases	ncRNAs	Encoded polypeptides/proteins	Functions	Mechanisms	References
Digestive system	ECSS	LINC00278	YY1BM	Inhibits the progression of ESCC	Inhibits the interaction between YY1 protein and androgen AR to reduce eEF2K expression	Wu et al^[^^49^^]^
	GC	lncAKR1C2	pep-AKR1C2	Promotes tube formation and migration of lymphatic endothelial cells and facilitates lymph node metastasis of GC	Regulates YAP phosphorylation to increase CPT1A expression and enhances fatty acid oxidation and ATP production	Zhu et al^[^^50^^]^
	GC	circRNA MTHFD2L	CM-248 aa	Inhibits the proliferation and metastasis of GC *in vitro* and *in vivo*	Targets the acidic structural domain of nuclear gene SET, and promotes dephosphorylation of AKT, extracellular signal-regulated kinase, and P65	Liu et al^[^^79^^]^
	GC	circPPP1R12A	circPPP1R12A-73 aa	Promotes the growth and metastasis of CC	Activate the Hippo-YAP signaling pathway	Zheng et al^[^^51^^]^
	HCC	lncRNA HBVPTPAP	Polypeptide	Induces apoptosis of HCC cells	Interacts with the intracellular domain of PILRA to activate the downstream JAK/STAT signaling pathway and initiate mitochondrial apoptotic pathway	Lun et al^[^^52^^]^
	iCCA	circRNA cGGNBP2	cGGNBP-184 aa	Promotes iCCA cell proliferation and metastasis	Interacts with STAT3 and enhances its phosphorylation at the Tyr705 site to form a positive feedback loop of IL-6/cGGNBP2-184aa/STAT3	Li et al^[^^53^^]^
	CRC	circATG4B	circATG4B-222 aa	Increases autophagy and decreases chemosensitivity of CRC cells	Interacts with TMED10 and prevents the binding of TMED10 to ATG4B	Pan et al^[^^54^^]^
Respiratory system	COPD	has-circ-0008833	circ-0008833-57 aa	Inhibits the proliferation of 16HBE and accelerate cellular pyroptosis	Promotes the expression of Caspase-1, IL-18, IL-1β, NLRP3 and GSDMD	Xie et al^[^^55^^]^
	Septic ALI	circCDC42	CDC42-165 aa	Exacerbates the pyroptosis of alveolar macrophages	Promotes the excessive activation of the Pyrin inflammasome	Xu et al^[^^56^^]^
	AAS	LncRNA Rsflos2	MPM-93	Promotes macrophage polarization to M2	Binds directly to Srsf-1	Zhang ^[^^57^^]^
	NPC	MIR-34A	MIR-34AHG-133 aa	Inhibits the proliferation and promotes the apoptosis of NPC cells	Regulates Ki-67 and Caspase-3 expression	Li ^[^^58^^]^
	NSCLC	AFAP1-AS1	ATMLP	Promotes malignant transformation and tumorigenesis of NSCLC	Disrupts mitochondrial autophagy through interaction with NIPSNAP1	Pei et al^[^^59^^]^
	Lung cancer	hsa_circRNA_103820	188-aa peptide	Reduces cell viability, facilitates apoptosis, and inhibits cell migration and invasion	Inhibits the AKT pathway	Zhou et al^[^^60^^]^
	LUAD	LINC00954	LINC00954-ORF	Inhibits cell growth, motility and invasion in A549 cells, and sensitizes PEM-resistant A549 cells to PEM	Unclear	Han et al^[^^61^^]^

ECSS: esophageal squamous cell carcinoma; AR: androgen receptor; GC: gastric cancer; AKT: protein kinase B; HCC: hepatocellular carcinoma; PILRA: paired immunoglobulin-like type 2 receptor alpha; iCCA: intrahepatic cholangiocarcinoma; STAT3: signal transduction and activator of transduction 3; CRC: colorectal cancer; TMED10: transmembrane P24 trafficking protein 10; ATG4B: Autophagy-related 4B cysteine peptidase; COPD: chronic obstructive pulmonary disease; HBE: human bronchial epithelial cells; IL: interleukin; NLRP3: NLR family pyrin domain containing 3; GSDMD: gasdermin D; ALI: acute lung injury; AAS: allergic asthma; Srsf-1: serine and arginine rich splicing factor 1; NPC: nasopharyngeal carcinoma; NSCLC: non-small cell lung cancer; NIPSNAP1: mitochondrial matrix proteins 4-nitrophenylphosphatase domain and nonneuronal SNAP25-like protein homolog 1; LUAD: lung adenocarcinoma; PEM: pemetrexed.

In hepatocellular carcinoma (HCC), Lun^52^ et al discovered that the cytoplasmic localization of the lncRNA HBVPTPAP enables the recruitment of ribosomal subunits via its IRES, leading to the translation of bioactive polypeptides. This peptide may induce apoptosis in HCC cells by interacting with the intracellular structural domain of the paired immunoglobulin-like type 2 receptor alpha (PILRA) to activate the downstream JAK/STAT signaling pathway and initiate the mitochondrial apoptotic pathway. In intrahepatic cholangiocarcinoma (iCCA), Li^53^ et al reported that a novel circRNA (cGGNBP2), induced by IL-6, can encode a protein of cGGNBP-184aa, which directly interacts with signal transduction and activator of transduction 3 (STAT3) and enhances its phosphorylation at the Tyr705 site. This interaction establishes a positive feedback loop of IL-6/cGGNBP2-184aa/STAT3, which promotes the proliferation and metastasis of iCCA cells. In colorectal cancer (CRC), Pan^54^ et al identified that circATG4B-222aa, a novel peptide encoded by exosomal circATG4B, competitively interacts with transmembrane P24 trafficking protein 10 (TMED10) and prevents the binding of TMED10 to autophagy-related 4B cysteine peptidase (ATG4B), thereby leading to an increase in autophagy and a reduction in the chemosensitivity of CRC cells.

These studies indicate that EDNEPs play a crucial role in either promoting or suppressing carcinogenesis across various gastrointestinal malignancies. In esophageal and gastric cancers, specific micropeptides influence tumor progression by regulating apoptosis, metabolic reprogramming, and lymphatic metastasis. In hepatocellular and colorectal cancers, specific micropeptides modulate tumor cell survival and drug resistance by targeting key mechanisms such as the JAK/STAT signaling pathway and autophagy. Collectively, these findings underscore the complex and diverse mechanisms of EDNEPs in the digestive system.

### Respiratory system

EDNEPs have been associated with various respiratory diseases, including chronic obstructive pulmonary disease (COPD), acute lung injury (ALI), and allergic asthma (AAS) (Table 2). Xie et al^55^ identified that hsa-circ-0008833 encodes a functional protein called circ-0008833-57aa, which accelerates the progression of COPD by promoting the expression of Caspase-1, interleukin (IL)-18, IL-1β, NLR family pyrin domain containing 3 (NLRP3) and Gasdermin D (GSDMD), thereby inhibiting the proliferation of human bronchial epithelial cells (16HBE) and accelerating cellular pyroptosis. In septic ALI, Xu et al^56^ found that exosomal circCDC42, originating from alveolar macrophages, encodes the CDC42-165aa protein, which promotes hyperactivation of the pyrin inflammasome and exacerbates pyroptosis in alveolar macrophages. In AAS, Zhang Y^57^ discovered that MPM-93, a functional short peptide encoded by lncRNA Rsflos2, is highly expressed in M2 macrophages and lung tissues of AAS mice. MPM-93 directly binds to serine and arginine rich splicing factor 1 (Srsf-1) and promotes M2 macrophage polarization. In contrast, the knockdown of Srsf-1 impairs the effect of MPM-93 on macrophages.

Furthermore, EDNEPs exert a significant impact on the progression of respiratory tumors. In nasopharyngeal carcinoma (NPC), Li^58^ found that HEK293 cell-derived exosomes contain the MIR-34A-encoded peptide (MIR-34AHG-133aa), and this peptide inhibits the proliferation and promotes apoptosis of NPC cells by regulating the nuclear proteins Ki-67 and Caspase-3. In non-small cell lung cancer (NSCLC), Pei et al^59^ demonstrated that the peptide ATMLP encoded by AFAP1-AS1 is upregulated in NSCLC tissues and disrupts mitochondrial autophagy by interacting with mitochondrial matrix proteins 4-nitrophenylphosphatase domain and nonneuronal SNAP25-like protein homolog 1 (NIPSNAP1), thereby promoting malignant transformation and tumorigenesis. In lung cancer, a 188-amino acid peptide derived from hsa_circRNA_103820 was found to exert potent tumor-suppressive effects, reducing cell viability, inducing apoptosis, and inhibiting cell migration and invasion.^60^ In lung adenocarcinoma (LUAD), LINC00954 encodes a novel polypeptide of LINC00954-ORF. Functionally, LINC00954-ORF represents a potential anticancer therapeutic for LUAD, possessing the capability to inhibit the growth, motility, and invasion of A549 cells and to sensitize pemetrexed (PEM)-resistant A549 cells to PEM.^61^

In summary, EDNEPs exert diverse functions in respiratory diseases by modulating key pathological processes. In inflammatory conditions (*e.g.*, COPD, ALI, asthma), they regulate inflammation and injury through mechanisms such as pyroptosis and macrophage polarization. In cancers (*e.g.*, nasopharyngeal carcinoma and lung cancer), they alter tumor progression and drug resistance by impacting cell proliferation, apoptosis, mitophagy, and chemotherapy sensitivity. These findings reveal the broad therapeutic potential of EDNEPs in the respiratory system.

### Urinary system

In the urinary system, EDNEPs have been investigated in renal cell carcinoma (RCC) and bladder cancer (BC) (Table 3). It has been shown that the micropeptide inhibiting actin cytoskeleton (MIAC), encoded by lncRNA ACO25154.2 within exosomes secreted by RCC cells, can directly bind to aquaporin 2 (AQP2), inhibit the activation of the EREG/EGFR signaling pathway, and modulate the activities of the PI3K/AKT and MAPK pathways, thereby inhibiting the proliferation and metastasis of RCC.^62^ Gu et al^63^ discovered that circGprc5a is upregulated in both stem cells and cancer tissues of BC and positively modulates the activity of BC stem cells. More importantly, the polypeptide encoded by circGprc5a interacts with the G protein-coupled receptor class C group 5 member A (Gprc5a) protein and is involved in the maintenance, self-renewal and metastasis of BC stem cells. These studies establish a contrasting role for EDNEPs: MIAC inhibits renal cell carcinoma progression, whereas the circGprc5a-encoded peptide drives malignancy in bladder cancer stem cells. Nevertheless, the field of EDNEPs in urinary tract tumors remains nascent and demands expanded study.

**Table 3:** The roles and mechanisms of EDNEPs in human diseases of the urinary, endocrine, reproductive, and circulatory systems.

Systems	Diseases	ncRNAs	Encoded polypeptides/proteins	Functions	Mechanisms	References
Urinary system	RCC	lncRNA ACO25154.2	MIAC	Inhibit the proliferation and metastasis of RCC	Binds to the AQP2 protein, inhibits the activation of the EREG/EGFR signaling pathway, and regulates the activities of the PI3K/AKT and MAPK pathways	Li et al^[^^62^^]^
	BC	circ-Gprc5a	Polypeptide	Regulates BC stem cell maintenance, self-renewal and metastasis	Interacts with the Gprc5a protein	Gu et al^[^^63^^]^
Endocrine and reproductive system	Diabetic retinal I/R injury	lncRNA 1810058124Rik	Stmp1	Protects retinal ganglionic cells from I/R injury	Decreases the activation of microglia and NLRP3 inflammasome pathways	Zheng et al^[^^64^^]^
	TNBC	LINC00665	CIP2A-BP	Inhibits the progression of TNBC	Binds to the tumor oncogene CIP2A, replaces the B56γ subunit of PP2A, releases the activity of PP2A, and inhibits the PI3K/AKT/NF-κB pathway	Guo et al^[^^33^^]^
	Endometrial dysfunction	lncRNA SNHG6	lncRNA SNHG6 ORF#2	Promotes human endometrial stromal and epithelial cell migration and epithelial–mesenchymal transformation	Activates the TGF-β/SMAD pathway	Zou et al^[^^65^^]^
	CC	circE7	E7 oncoprotein	Promotes the growth of CC cells *in vitro* and xenograft tumors *in vivo*	Unclear	Zhao et al^[^^66^^]^
	OC	lncRNA CTBP1-DT	DDUP	Promotes DNA damage repair and cisplatin resistance of OC cells	Interacts with histone H2A	Yu et al^[^^67^^]^
Circulatory system	Cardiac remodeling	circ0036176	Myo9a-208	Myo9a-208 mediates the inhibitory effect of circ_0036176 on cardiac fibroblast proliferation	Inhibits the cyclin/Rb signaling pathway	Guo et al^[^^68^^]^
	Myocardial fibrosis	lncPostn	CFAP	Inhibits the proliferation and activation of myocardial fibroblasts, reduces myocardial fibrosis and improves cardiac function	Unclear	Wang ^[^^69^^]^
	–	muscle-specific lncRNA	DWORF	Enhances SERCA activity	Replaces the SERCA inhibitors phospholipids, myolipids, and myosin	Nelson et al ^[^^70^^]^
	Cardiomyopathy	miRNA	miRNA-related N-exo peptides	Inhibits the secretion of DMD-exo both *in vitro* and *in vivo*, and improves stress vulnerability	Unclear	Gartz et al ^[^^72^^]^

RCC: renal cell carcinoma; MIAC: micropeptide inhibiting actin cytoskeleton; AQP2: Aquaporin 2; BC: bladder cancer; Gprc5a: G protein-coupled receptor class C group 5 member A; I/R: ischemia/reperfusion; TNBC: triple-negative breast cancer; TGF-β: transforming growth factor-β; CC: cervical cancer; OC: ovarian cancer; SERCA: sarcoplasmic/endoplasmic reticulum Ca ^2+^-ATPase.

### Endocrine and reproductive system

Research on EDNEPs in the endocrine system has primarily focused on diabetic complications and breast cancer (Table 3). In diabetic retinal ischemia/reperfusion (I/R) injury, Zheng et al^64^ identified that lncRNA 1810058124Rik encodes a mitochondrion-localized micropeptide, Stmp1, which protects retinal ganglionic cells from I/R injury by reducing the activation of microglia and the NLRP3 inflammasome pathways, indicating that Stmp1 is a novel therapeutic target for diabetic retinopathy. In triple-negative breast cancer (TNBC), Guo et al^33^ discovered that the micropeptide CIP2A-BP encoded by LINC00665 is regulated by transforming growth factor-β (TGF-β). Mechanistically, CIP2A-BP directly interacts with oncogenic CIP2A and replaces the B56γ subunit of PP2A, which restores PP2A activity and inhibits the PI3K/AKT/NF-κB pathway. CIP2A-BP suppresses TNBC progression and represents a potential therapeutic for TNBC metastasis.

In the female reproductive system, a study by Zou et al^65^ revealed that a small peptide translated from the lncRNA SNHG6 ORF#2 promotes human endometrial stromal and epithelial cell migration and epithelial–mesenchymal transformation through activation of the TGF-β/SMAD signaling pathway. In cervical cancer (CC), human papillomavirus type 16 (HPV16)-infected cells release exosomes containing circE7, which harbors the intact E7 ORF that encodes the E7 oncoprotein mediated by m6A modification.^66^ Further experiments confirmed that knockdown of circE7 or interference with m6A modification could reduce the expression of the E7 oncoprotein and inhibit the growth of tumor cells *in vitro* as well as xenograft tumors *in vivo*. Additionally, the peptide DDUP, encoded by the lncRNA CTBP1-DT, enhances DNA damage repair and cisplatin resistance in ovarian cancer cells by interacting with histone H2A.^67^

Collectively, EDNEPs influence pathophysiology through diverse mechanisms in the endocrine and reproductive systems. Stmp1 is neuroprotective in diabetic retinopathy, while CIP2A-BP acts as a tumor suppressor in breast cancer by restoring PP2A activity. In female reproductive tumors, however, specific micropeptides drive malignancy and drug resistance by activating oncogenic signaling, encoding viral oncoproteins, or enhancing DNA repair. These findings reveal the significant potential of EDNEPs as novel biomarkers and therapeutic targets in the endocrine and reproductive systems.

### Circulatory system

The functions of EDNEPs in heart diseases have become increasingly clear in recent years (Table 3). In cardiac remodeling, Guo et al^68^ discovered that exosome-derived circ_0036176 encodes the Myo9a-208 protein, which is 208 aa in length due to the presence of an IRES element and a 627 nt ORF, and miR-218-5p represses Myo9a-208 expression by binding to circ_0036176. Cellular function assays demonstrated that Myo9a-208 mediates the inhibitory effect of circ_0036176 on the proliferation of cardiac fibroblasts by inhibiting the cyclin/Rb signaling pathway. In myocardial fibrosis, lncPostn encodes a bioactive polypeptide CFAP with a length of 92 aa, which inhibits the proliferation and activation of myocardial fibroblasts, attenuates myocardial fibrosis and improves cardiac function.^69^ In 2016, Nelson et al^70^ identified a muscle-specific lncRNA that encodes a polypeptide, DWORF, which is 34 aa in length. DWORF enhances SERCA activity by replacing the sarcoplasmic/endoplasmic reticulum Ca^2+^-ATPase (SERCA) inhibitors of phospholipids, myolipids, and myosin and is the only known endogenous peptide that physically activates the SERCA pump. Another study by Morales^71^ et al reported that DWORF deficiency leads to SERCA dysfunction in mdx mice, and DWORF gene therapy shows potential for treating cardiomyopathy associated with Duchenne muscular dystrophy. In cardiomyopathy, Gartz et al^72^ discovered that miRNA-related N-exo peptides derived from exosomes inhibited DMD-exo secretion both *in vitro* and *in vivo*, thereby improving stress vulnerability.

In summary, EDNEPs regulate heart diseases through diverse mechanisms. They inhibit pathological processes such as myocardial fibrosis via peptides such as Myo9a-208 and CFAP and enhance cardiac function by improving calcium cycling via DWORF. These findings not only elucidate the critical role of EDNEPs in cardiac remodeling, fibrosis, and cardiomyopathy but also reveal novel potential targets and therapeutic strategies for heart disease.

### Immune system

Current studies on EDNEPs in the immune system have focused on immune diseases and tumor immunity (Table 4). Niu et al^25^ discovered that the lncRNA MIR155HG encodes a micropeptide, miPEP155, which regulates MHC–II–mediated antigen presentation and T-cell activation, alters T-cell polarization status, and ameliorates autoimmune inflammation by disrupting the interaction of HSC70-HSP90. In addition, Bhatta et al^73^ discovered that the mitochondrial micropeptide Mm47 with a length of 47 aa, encoded by the lncRNA 1810058I24Rik, can mediate the activation of the NLRP3 inflammasome, which leads to Caspase-1 activation and IL-1β secretion, suggesting an important role of Mm47 in innate immunity. In tumor immunity, a previous study demonstrated that lncRNA meloe in melanoma cells produces immunogenic MELOE antigen via IRES-dependent translation. Recently, Charpentier et al^74^ confirmed that the lncRNA meloe encodes the polypeptide MELOE-1 and provided evidence that heterogenic nuclear ribonucleoprotein A1 (hnRNP-A1) binds to the MELOE-1 IRES and functions as an IRES transactivator (ITAF) to promote the translation of MELOE-1 in melanoma cells. Lipoic acid-induced endoplasmic reticulum stress promotes the translocation of hnRNP-A1 and enhances the translation of MELOE-1 and the recognition of melanoma cells by T lymphocytes. Furthermore, Zhou et al^27^ discovered that miPEP31, encoded by pri-miRNA-31, inhibits autoimmunity by promoting Treg differentiation.

**Table 4:** The roles and mechanisms of EDNEPs in human diseases of the immune and nervous systems.

Systems	Diseases	ncRNAs	Encoded polypeptides/proteins	Functions	Mechanisms	References
Immune system	Autoimmune inflammation	lncRNA MIR155HG	miPEP155	Regulates MHC–II–mediated antigen presentation and T-cell activation, alters T-cell polarization status, and ameliorates autoimmune inflammation	Disrupts the interaction of HSC70-HSP90	Niu et al ^[^^25^^]^
	Innate immunity	lncRNA 1810058I24Rik	Mm47	Plays an important role in innate immunity	Regulates NLRP3-dependent Caspase-1 activation and IL-1β secretion	Bhatta et al ^[^^73^^]^
	Tumor immunity	lncRNA meloe	MELOE-1	Enhances the recognition of melanoma cells by T lymphocytes	Unclear	Charpentier et al ^[^^74^^]^
	Autoimmunity	pri-miRNA-31	miPEP31	Represses autoimmunity	Promotes Treg differentiation	Zhou et al ^[^^27^^]^
Nervous system	GBM	LINC02381	micropeptide	Regulates ferroptosis	Binds with SLC2A10	Jiang et al ^[^^75^^]^
	GBM	circ-FBXW7	FBXW7-185aa	Inhibits the occurrence and progression of GBM	Promotes the degradation of proto-oncogene c-Myc by competitively binding to the ubiquitination protein USP28	Yang et al ^[^^38^^]^
	GBM	circ-AKT3	AKT-174aa	Reduces proliferation, radiation resistance and tumorigenicity of GBM	Binds with p-PDK1 to reduce phosphorylation of AKt-Thr308 and inhibits the PI3K/AKT signaling pathway	Xia et al ^[^^76^^]^
	GBM	LINC-PINT	PINT87aa	Inhibits the proliferation of GBM *in vitro* and *in vivo*	Interacts with the PAF1c complex to inhibit transcriptional elongation of multiple oncogenes	Zhang et al ^[^^40^^]^
	NB	HNF4A-AS1	sPEP1	Promotes the self-renewal and metastasis of NB stem cells	Interacts with eEF1A1 and inhibits trans-activation of SMAD4	Song et al ^[^^77^^]^

MHC-II: major histocompatibility complex class II; GBM: glioblastoma; LINC-PINT: long intergenic nonprotein coding RNA p53 induced transcript; PAF1c: polymerase-associated factor 1 complex; NB: neuroblastoma; sPEP1: small 51 aa peptide; eEF1A1: eukaryotic translation extension factor 1α1; SMAD4: SMAD family member 4.

In summary, EDNEPs play a dual regulatory role in the immune system. They modulate autoimmune pathology by regulating antigen presentation and inflammasome activity, while in oncology, they can be processed into immunogenic antigens that potentiate T-cell recognition of malignancies. These insights establish EDNEPs as pivotal immune regulators, paving the way for novel therapeutic strategies against immune-related diseases.

### Nervous system

In the nervous system, EDNEPs significantly impact brain tumor progression (Table 4). Jiang et al^75^ found that LINC02381 is highly expressed in glioblastoma (GBM), its encoded micropeptide is located in exosomes, and this polypeptide may regulate ferroptosis by binding to solute carrier family 2 member 10 (SLC2A10) through machine learning. Yang et al^38^ identified that circ-FBXW7 encodes a novel 21-kDa protein, FBXW7-185aa, which promotes the degradation of the proto-oncogene c-Myc by competitively binding to the ubiquitination protein USP28, thereby inhibiting the onset and progression of GBM. Low expression of circ-AKT3 can encode a novel protein, AKT-174aa, by overlapping start-stop codons. Mechanistically, AKT-174aa competitively binds to p-PDK1 to reduce phosphorylation of Akt-Thr308 and inhibit the PI3K/AKT signaling pathway, leading to decreased proliferation, radiation resistance and tumorigenicity of GBM.^76^ In addition, Zhang et al^40^ found that a circRNA originating from the long intergenic nonprotein coding RNA p53-induced transcript (LINC-PINT) can encode the polypeptide PINT87aa, which directly interacts with the polymerase-associated factor 1 complex (PAF1c) to inhibit the transcriptional elongation of multiple oncogenes, thereby suppressing the proliferation of GBM both *in vitro* and *in vivo*. In neuroblastoma (NB), hepatocyte nuclear factor 4 alpha antisense RNA 1 (HNF4A-AS1) encodes a small 51-aa peptide (sPEP1) that directly interacts with eukaryotic translation extension factor 1α1 (eEF1A1), inhibits transactivation of SMAD family member 4 (SMAD4), and enhances the transcription of stem cell genes associated with tumor progression, leading to self-renewal and metastasis of NB stem cells.^77^

In conclusion, in GBM, functional peptides encoded by circRNAs and lncRNAs suppress tumor progression and increase radiotherapy sensitivity by degrading c-Myc and inhibiting the PI3K/AKT pathway. In contrast, specific micropeptides in NB promote tumor stemness by regulating transcription elongation and stem cell gene expression. Collectively, these findings highlight the potential of EDNEPs as novel therapeutic targets for brain tumors.

### Clinical applications of EDNEPs

#### *Biomarkers*

EDNEPs exhibit distinct expression patterns in diseases of various systems, and detection of EDNEPs may facilitate early diagnosis of diseases. For instance, the expression of circZKSAaa, a peptide derived from circZKSCAN1, is significantly reduced in HCC tumor tissues compared with paired normal tissues, and receiver operating characteristic (ROC) analysis demonstrated 100% diagnostic specificity of circZKSCAN1 for HCC, suggesting that circZKSAaa may serve as a diagnostic biomarker for HCC. Furthermore, in clinical studies, it has been demonstrated that circZKSaa, as a potential prognostic biomarker for HCC, can make HCC cells sensitive to sorafenib treatment, and circZKSaa can enhance the antitumor effect of sorafenib in HCC cells.^78^ The expression levels of EDNEPs are correlated with the severity of disease, the responses to treatment or the survival of the patients and can serve as biomarkers for disease prognosis, where changes in these peptides can be detected to make an assessment of the prognosis of the patient and to make timely adjustments in the treatment regime. For example, in GC patients, decreased expression of the circ-MTHFD2L-encoded peptide CM-248aa is associated with higher TNM stage and histopathological grade, making it a potential prognostic biomarker for GC. Additionally, CM-248aa has been identified as a novel clinical tumor suppressor and an endogenous inhibitor that interacts with SET-PP2A, negatively regulating the PI3K-AKT, MAPK, and NF-κB pathways to inhibit the progression of GC.^79^ The upregulated expression of the splicing-regulated small protein (SRSP) encoded by LOC90024 is positively associated with malignant phenotypes and poor prognosis in patients with CRC.^80^

### Therapeutic agents

In the field of modern medicine, exosomes have demonstrated significant potential as carriers for disease treatment, and EDNEPs can function as specific signaling molecules to directly interact with drug transporters and influence cellular behavior to facilitate disease treatment. Exosomes can protect EDNEPs from the complex *in vivo* environment and precisely deliver them into target cells for therapeutic purposes. Currently, tumor therapy in animal models involves using exosomes to transport EDNEPs that inhibit tumor development or to knock down or modify peptides that promote tumor growth. Currently, the excellent therapeutic efficacy of EDNEPs delivered by exosomes has been demonstrated in animal models of tumors. Chang et al^81^ found that the SHPRH-146aa protein encoded by circ-SHPRH regulates the P21 cell cycle pathway, and overexpression of SHPRH-146aa effectively inhibits the growth of NB tumors in mice, which is more effective in combination with everolimus. In addition, Liu et al^82^ discovered that circ-EGFR encodes a novel epidermal growth factor receptor variant (rtEGFR), and silencing rtEGFR in GBM cells attenuates tumorigenicity and enhances the antitumor effect.

### Therapeutic targets

EDNEPs can interact with key molecules in critical signaling pathways of diseases, such as binding to kinases, phosphatases, or transcription factors, thereby altering the activity or function of these molecules. Therefore, inhibition or activation of these peptides to modulate the activity of the key signaling pathways represents a potential therapeutic strategy for diseases.^83^ Laumont et al discovered that most tumor-specific antigens are derived from ncRNAs and that EDNEPs are promising targets for cancer immunotherapy.^84^ In addition, the peptides encoded by circPINT and circAKT3 in GBM could also serve as potential therapeutic targets.^85^^,^^86^ Furthermore, the novel HER2 variant HER2-103, generated by circHER2 translation, can continuously activate AKT by binding to the CRI structure of HER2, promoting the proliferation and invasion of TNBC cells and causing tumorigenesis. Pertuzumab (an anti-HER2 antibody) is ineffective for HER2-negative TNBC patients. However, TNBC cells expressing HER2-103 and mouse xenografts are sensitive to pertuzumab treatment by targeting the shared CRI domain of HER2, making it the best target for pertuzumab (or in combination with trastuzumab) treatment in TNBC.^87^^,^^88^

## Conclusions and prospects

In recent years, the functions of ncRNAs and ncRNA-encoded peptides have been favored for their functions in diseases of various systems. Exosomes are more widely present in blood and body fluids and function as carriers to transmit intercellular communication by carrying and delivering specific signaling molecules. EDNEPs can be used as biomarkers for the diagnosis and prognosis of diseases, especially in tumors, which provides a new direction for disease diagnosis and treatment. Meanwhile, EDNEPs can be used as potential therapeutic targets for diseases or delivered to specific organs and tissues as therapeutic agents, providing new strategies for disease treatment. Currently, the study and application of EDNEPs are mainly focused on the field of oncology, with less research on nontumor diseases. Although the potential roles of EDNEPs in tumor progression have been discovered, their regulatory mechanisms, especially how these peptides interact with other signaling molecules, are still poorly explored. Furthermore, although exosomes are widely present in plasma, serum and urine, they have low abundance and small size, and the existing technologies and methods are prone to damaging exosomes. This makes it extremely difficult to extract and separate them to obtain highly pure and abundant exosomes, which also indirectly limits the clinical application of these peptides. Furthermore, EDNEPs may also cause relevant immune responses or other adverse reactions, and thus, safety issues need to be further verified.

To solve the above problems, first, research on the functions and regulatory mechanisms of EDNEPs should be strengthened to reveal their unique mode of action and provide a solid theoretical foundation for clinical practical application. For instance, through high-precision interaction molecule screening and verification techniques to confirm the specificity of interactions, thereby uncovering the regulation of metabolic pathways by EDNEPs; verifying nonclassical translation mechanisms (such as IRES-mediated and cap-independent translation), directly demonstrating the translation process; and using live imaging technology to monitor the dynamic interactions between EDNEPs and target proteins in living cells in real time, addressing the issue that “static detection cannot reflect dynamic regulation”. Second, it is necessary to improve exosome separation and purification technology to obtain high-purity and high-yield exosomes to meet experimental and clinical needs. For example, regarding the low purity issue of the polyethylene glycol (PEG) precipitation method, “immunoprecipitation magnetic beads” can be used to remove contaminating proteins, thereby increasing the purity of exosomes by 3–5 times. Through microfluidic chip technology, exosomes can be rapidly isolated from small samples with high purity, and their activity is retained. In addition, pH separation technology utilizes the specific isoelectric point of the surface proteins of exosomes, meaning that at a specific pH value, the net charge of the protein is zero. By adjusting the pH value of the solution, the charge of the surface proteins of exosomes changes, thereby achieving the separation of exosomes. This avoids the damage of exosomes caused by high-intensity mechanical force or chemical treatment. Compared with traditional methods such as ultracentrifugation and PEG precipitation, these technologies are not only simple and convenient but also retain the integrity and functionality of exosomes and can efficiently extract exosomes from various biological samples. Finally, rational preclinical trials (such as immunogenicity testing and toxicity assessment) and clinical trials (such as disease model validation and dose-effect relationship verification) should be conducted to verify the safety and efficacy of EDNEPs as diagnostic and prognostic markers and potential therapeutic agents and targets for diseases. With technological breakthroughs and interdisciplinary integration, in the future, we need to achieve a transformation from “experience-driven” to “data-driven”, integrating AI technology, single-cell technology and nanotechnology into the research scope of EDNEPs to improve work efficiency and reduce the blindness of experiments. Although 90% of EDNEP research is concentrated in oncology, it is imperative to diversify its scope to nononcological diseases, including infectious, inflammatory, autoimmune, degenerative, metabolic, and genetic disorders. Moreover, the application of EDNEPs should evolve from a “single biomarker/target” approach to a multidimensional model that integrates diagnosis, monitoring, and treatment, thereby offering new insights and targets for disease management.

## CRediT authorship contribution statement

**Xinyuan Feng:** Writing – original draft, Software, Methodology, Investigation, Formal analysis, Conceptualization. **Meihuan Chen:** Writing – original draft, Data curation. **Dahang Ye:** Visualization, Investigation. **Ying Zhu:** Validation, Software. **Liangpu Xu:** Visualization. **Hailong Huang:** Writing – review & editing, Supervision, Resources, Funding acquisition, Conceptualization.

## Funding

This project was supported by the Major Scientific Research Program for Young and Middle-aged Health Professionals of Fujian Province (China) (No. 2023ZQNZD009), the National Natural Science Foundation of China (No. 81970170), the Joint Funds for the Innovation of Science and Technology of Fujian province (China) (No. 2023Y9364), and Fujian Provincial Natural Science Foundation of China (No. 2023J011217, 2025J011152, 2025J011153).

## Conflict of interests

The author declares no conflict of interests.
